# The effects of an aerobic training intervention on cognition, grey matter volumes and white matter microstructure

**DOI:** 10.1016/j.physbeh.2020.112923

**Published:** 2020-09-01

**Authors:** Claire E. Sexton, Jill F. Betts, Andrea Dennis, Aiden Doherty, Paul Leeson, Cameron Holloway, Erica Dall'Armellina, Anderson M. Winkler, Naiara Demnitz, Thomas Wassenaar, Helen Dawes, Heidi Johansen-Berg

**Affiliations:** a FMRIB Centre, Wellcome Centre for Integrative Neuroimaging, Nuffield Department of Clinical Neurosciences, University of Oxford, Oxford, UK, OX3 9DU; b Oxford Centre for Human Brain Activity, Wellcome Centre for Integrative Neuroimaging, Department of Psychiatry, University of Oxford, UK; c Nuffield Department of Population Health, University of Oxford, Oxford, UK, OX3 7LF; d Cardiovascular Clinical Research Facility, Division of Cardiovascular Medicine, University of Oxford, Oxford, UK, OX3 9DU; e University of Oxford Centre for Clinical Magnetic Resonance, University of Oxford, Oxford, UK, OX3 9DU; f Department of Psychiatry, University of Oxford, Oxford, UK, OX3 7JX; g Faculty of Health and Life Sciences, Oxford Brookes University, Oxford, UK, OX3 0BP

**Keywords:** Aging, Cardiorespiratory fitness, Diffusion tensor imaging, Magnetic resonance imaging, Physical activity, White matter

## Abstract

While there is strong evidence from observational studies that physical activity is associated with reduced risk of cognitive decline and dementia, the extent to which aerobic training interventions impact on cognitive health and brain structure remains subject to debate. In a pilot study of 46 healthy older adults (66.6 years ± 5.2 years, 63% female), we compared the effects of a twelve-week aerobic training programme to a waitlist control condition on cardiorespiratory fitness, cognition and magnetic resonance imaging (MRI) outcomes. Cardiorespiratory fitness was assessed by VO_2_ max testing. Cognitive assessments spanned executive function, memory and processing speed. Structural MRI analysis included examination of hippocampal volume, and voxel-wise assessment of grey matter volumes using voxel-based morphometry. Diffusion tensor imaging analysis of fractional anisotropy, axial diffusivity and radial diffusivity was performed using tract-based spatial statistics. While the intervention successfully increased cardiorespiratory fitness, there was no evidence that the aerobic training programme led to changes in cognitive functioning or measures of brain structure in older adults. Interventions that are longer lasting, multi-factorial, or targeted at specific high-risk populations, may yield more encouraging results.

## Highlights

• Aerobic exercise has been proposed to improve cognitive health, via brain structure.
• 12-weeks of aerobic exercise did not change cognitive, grey or white matter measures.
• Interventions may need to be longer lasting or multifactorial.

## 1 Introduction

Across animal and human studies, it has been widely proposed that higher levels of physical activity (PA) and cardiorespiratory fitness (CRF) can promote successful cognitive aging [1,2], with reviews of observational studies consistently concluding that higher levels of PA are associated with a reduced risk of cognitive decline and dementia [3], [4], [5]. The current picture from reviews of randomized-controlled trials (RCTs) examining the effects of aerobic training programmes on cognition in healthy older adults, however, is mixed. Although several meta-analyses have reported that aerobic training programmes are associated with improvements across multiple cognitive domains [6], [7], [8], [9], others have concluded that there is *no* evidence that aerobic training programmes have any benefit in cognitively healthy older adults [10,11]. Variation in results is likely to stem from differences in the inclusion criteria with regard to publication date, training programme employed, and the baseline age and cognitive status of participants. Heterogeneity within and between studies will also be compounded by limitations associated with cognitive testing, as performance is influenced by a number of factors, including practice effects and participant effort.

The objective nature of structural MRI markers means they may provide more sensitive measures of cognitive health and increase consistency in results. Indeed, a number of observational magnetic resonance imaging (MRI) studies have linked higher levels of PA and CRF to greater grey matter volumes including the prefrontal cortex and hippocampus [12], [13], [14]. In addition, higher levels of PA and CRF have also been associated with markers of white matter microstructure, both globally and within the frontal lobe [15]. To date, though, the results of interventional studies examining the effects of aerobic training programmes on brain structure have been variable.

With regard to interventional studies of older adults examining grey matter volumes, a meta-analysis of six studies examining the effect of aerobic PA on hippocampal volume in healthy older adults found that aerobic PA did not significantly increase total hippocampal volume compared with control conditions [16]. However, a meta-analysis of the five studies to report lateralised results showed statistically significant positive effect of exercise on both left and right hippocampal volumes [16]. Further, in analyses that grouped participants assigned to either the aerobic training programme or the control intervention, change in fitness has been positively associated with change in hippocampal volume [17], [18], [19] and hippocampal volume post-intervention [20]. There have been similarly mixed results for studies examining the prefrontal cortex [18,21,22].

There have been fewer interventional studies of older adults examining white matter microstructure, with no significant differences between control and intervention groups found for fractional anisotropy (FA), axial diffusivity (AD) or radial diffusivity (RD) in studies examining a ten-week multi-modal physical training programme [23], a six-month [24] aerobic walking programme, or a year-long [25] aerobic walking programmes. However, greater percentage change in fitness has been associated with significant increases in prefrontal, parietal and temporal FA within the aerobic training group in one study [25].

While RCT analyses of cognitive, grey matter, and white matter measures in have been reported in overlapping samples of differing sizes in independent publications [17,25], to the best of our knowledge, the effect of an PA intervention across cognitive, grey matter, and white matter measures has not been concurrently reported in a single sample of healthy older adults. Concurrent analyses may indicate whether results across modalities are complimentary, and could, for example, identify a *‘hippocampal signature’* characterised by increased hippocampal volume, increased FA and reduced FA in hippocampal tracts, and improved performance on tests of episodic memory, or a *‘frontal signature’* characterised by increased volume within the prefrontal cortex, increased FA and reduced RD in prefrontal tracts, and improved performance in tests of executive functioning.

Here, we report the results from the Cognitive Health in Ageing Exercise Study, a pilot study that examined the effects of an aerobic training intervention on executive function, memory and processing speed, and both global and voxel-wise measures of grey matter volume and white matter microstructure. Tentatively, we hypothesise that, compared with a control group, the aerobic training group will show improvements in cognitive performance, increases in grey matter volumes and improved white matter microstructure, with results localised to frontal or hippocampal regions. Further, we hypothesise that change in fitness over the course of the study will be positively associated with change in cognitive and MRI markers.

## 2 Methods

### 2.1 Inclusion and exclusion criteria

Ethical approval was obtained from the Local Research Ethics Committee (Oxford REC B Ref 10/H0605/48), informed written consent was obtained from all participants, and all research was performed in accordance with the relevant guidelines and regulations.

Potential volunteers were recruited from the local community through advertisements and word-of-mouth, and screened over the telephone to ensure they were aged between 60-85 years, self-reported that they participated in fewer than 60 minutes per week of PA sufficient to raise their heart rate (participants were asked “Do you do currently do any exercise, such as swimming or any fitness classes?” and “What other physical activity do you currently do during a typical week, such as walking, gardening, or housework?”, before the single-item question “How many minutes a week do you do activity that raises your heart rate?”), had no known contraindications to MRI scanning or fitness testing (assessed using the physical activity readiness questionnaire, PAR-Q; participants with a history of major vasculature problems or on heart rate-controlling medications were excluded), reported no history or current investigation of a neurological disorder, reported no symptoms or treatment for a psychiatric illness within the past year, and believed they would be able to commit to the requirements of study.

Eligible participants were invited to attend a screening assessment, which included administration of the Mini-Mental State Examination (MMSE) to ensure participants did not display signs of cognitive impairment (MMSE ≤ 26), and Structured Clinical Interview for DSM-IV-TR Axis I Disorders, Research Version, Non-Patient Edition (SCID-I/NP) [26] to ensure participants had not displayed clinically significant symptoms of a psychiatric illness within the past year. Blood pressure was measured three times in a supine position (GE V100 Dinamaps; GE Healthcare, Hatfield, Herts.) before a resting electrocardiogram (ECG) was administered. If the researcher and technician identified no contraindications, an exercise stress test was then administered, which was subsequently reviewed by a cardiologist.

### 2.2 Overview of study design

Following baseline assessments (timepoint 1, including fitness, PA, cognitive and MRI assessments), participants were randomly allocated to the PA intervention or a control group. Participants assigned to the PA programme were asked to attend monitored stationary cycling sessions three days a week, for 12 consecutive weeks. After 12-weeks, participants were invited for follow-up assessments (timepoint 2, again including fitness, PA, cognitive and MRI assessments).

### 2.3 Randomization and blinding

Participants were stratified into three age groups (60-69 years, 70-79 years and 80-84 years) after their baseline assessments, and randomly allocated to the PA intervention or a control group, according to a list of computer-generated random numbers in blocks of 4 (2 persons randomly allocated to each group) [27]. Allocation numbers were kept in sequentially numbered, opaque, sealed envelopes prepared by a researcher not directly involved in the recruitment or assessment of participants. It was not feasible for participants or intervention supervisors to be blind to group allocation. The primary researcher responsible for administering cognitive and physical fitness assessments remained blind to group allocation throughout the study.

### 2.4 Intervention

Participants assigned to the PA programme were asked to attend monitored training sessions three days a week, for 12 consecutive weeks. The training consisted of 30 minutes continuous cycling at a cadence between 60-70 Revolutions Per Minute (RPM), on an upright exercise bike, and to maintain a heart rate consistent with their aerobic training zone of between 55%-85% of their maximum HR. Training zones were calculated using age-predicted heart rate maximum (220 – age) [28] and were monitored by an experimenter every 5 minutes using digital heart rate monitoring hand sensors. Participants started at the lower end of the aerobic zone for the first few sessions and were encouraged to progress by increasing the heart rate range as the weeks progressed by increasing the resistance. Heart rate was allowed to increase through the programme but maintained within the aerobic training range as the weeks progressed. Participants assigned to the control group were asked to continue with their normal everyday routine and not to begin a PA programme.

### 2.5 Demographics

Age, sex and education level was recorded for all participants. Education level was scored on a five-point scale: (1) no qualifications, (2) O levels or equivalent, (3) A levels, college certificate or professional qualification, (4) degree, (5) higher degree [29].

### 2.6 Cognitive assessments

At baseline and follow-up, participants completed a battery of cognitive tests, comprising both computerised and pen-and-paper tasks, which were subsequently divided into the domains of executive function, memory and processing speed. The executive function domain included digit span: forward, backward and sequence [30], fluency: letter and category, trail-making test (TMT): B [31], COGSTATE® (www.cogstate.com) one-back, and COGSTATE® two-back. The memory domain included Hopkins Verbal Learning Test Revised (HVLT-R): total recall, delayed recall and recognition [32], Rey-Osterrieth Complex Figure (RCF): immediate recall, delayed recall and recognition [33], and COGSTATE® continuous paired associate learning errors. The processing speed domain included TMT: A [31], digit coding [30], and Cambridge Neuropsychological Test Automated Battery Reaction Time touchscreen task (CANTAB RTI; CANTABeclipse 5.0; Cambridge Cognition Ltd): median simple reaction time, median choice reaction time, median simple movement time, median choice movement time [34], COGSTATE® detection task speed and COGSTATE® identification task speed.

Where necessary, signs were reversed to ensure that higher scores represented a better performance for all variables (e.g. TMT, CANTAB: reaction time task, COGSTATE® continuous paired associate learning, detection and identification tasks). Alternative versions of the letter fluency (first assessment F, second assessment S, third assessment B) and HVLT-R tests were administered at repeat assessments [32,35].

### 2.7 Cardiorespiratory fitness assessment

CRF was assessed using a VO_2_ max test, which measures the volume of oxygen utilized by the body per unit of time at the peak of physical exertion. The VO_2_ max testing procedure was conducted on a cycle ergometer (Monarch, Sweden). The continuous, incremental test started with a warm-up phase of 3 minutes at 0 Watts (W) and then increased to 25 W for 2 minutes. Thereafter, resistance was increased by 25 W every 2 minutes. The participants were instructed to maintain a cadence of 60 revolutions per minute (RPM) throughout. The test was ended when volitional exhaustion was reached or the participant was no longer able to maintain a pedal rate of 60 RPM.

Oxygen consumption (VO_2_) and heart rate (HR) were measured throughout the test and Rating of Perceived Exertion (RPE) was recorded prior to each increment using the Borg CR10, 0-10 scale (Borg, 1970). HR was measured using a Polar Heart Rate Monitor (Polar, Finland) and oxygen consumption was assessed using a fully automated indirect calorimetry system (Metalyser, Cortex), sampling expired air collected through a face-mask with every breath. The calorimetry system was calibrated prior to each test using standard calibration gas (15% O_2_ and 5% CO_2_, balance N_2_) and atmospheric pressure and volume according the manufacturer's instructions. VO_2_ data were smoothed using a 9-breath moving average. In addition, data were time-averaged in 5-second intervals.

Maximum oxygen consumption was calculated as the largest 30 second average and was assessed by the attainment of the following criteria (a) maximal respiratory exchange ratio (RER) ≥ 1.1 (met in 93% of all tests), (b) maximal heart rate within 10 beats/min age-predicted maximum (220-age) (met in 72% of all tests), (c) a plateau in VO_2_ with increase in external work (met in 7% of all tests), (d) final RPE reported as maximal (met in 11% of all tests). On average, participants met 1.8 of the four criteria for maximum oxygen consumption, so results are referred to as VO_2_ peak, rather than VO_2_ max.

### 2.8 Physical activity assessments

PA was assessed subjectively using the CHAMPS questionnaire [36]. The CHAMPS questionnaire is a 41-item self-administered questionnaire, designed for older adults, in which participants report the frequency and duration of various activities in a typical week in the past four weeks, with each activity assigned a Metabolic Equivalent of Task (MET) value. MET.Minutes per week for moderate-to-vigorous PA was calculated from 20 items with metabolic equivalents MET ≥ 3.0.

PA was assessed objectively at each time-point using a wrist-worn accelerometer (GENEActiv®) instructed to be worn on the non-dominant hand for nine consecutive days. The accelerometer was sampled at 87.5 Hz and data were stored in gravity units (1g = 9.81 m/s^2^) for 3 axes, with no action needed from participants. Accelerometry data were processed following the methods developed by the UK Biobank Expert Working Group [37], described in full in the Supplementary Material – Text S1, and a PA outcome variable, average vector magnitude, was constructed.

### 2.9 MRI

MRI data were acquired at the Oxford Centre for Functional MRI of the Brain (FMRIB) using a 3-Tesla, Siemens Magnetom Verio (Erlangen, Germany) scanner with 32-channel head coil. All image analysis was performed using tools from the FMRIB Software Library (FSL, version 5.0; http://www.fmrib.ox.ac.uk/fsl).

T1-weighted structural images were acquired using a three- dimensional rapid gradient echo sequence with repetition time 2530ms, echo time 7.37ms, flip angle 7°, field of view 256mm and voxel dimensions 1.0mm isotropic. T1-weighted images were processed using fsl_anat (http://fsl.fmrib.ox.ac.uk/fsl/fslwiki/fsl_ anat). Partial-volume tissue segmentation was performed using the FMRIB Automated Segmentation Tool (FAST) [38]. Whole brain volume was obtained by summing the volumes of grey matter, white matter and cerebrospinal fluid. Hippocampal volume was calculated using FMRIB Integrated Registration and Segmentation Tool (FIRST) [39]. Three-stage affine registration was used, and all volumes were manually checked and edited, where necessary, by a researcher blind to group allocation. Specifically, images were checked and edited so that (i) voxels primarily comprised of cerebrospinal fluid were excluded, (ii) voxels encompassing the fimbria were included, (iii) voxels extending into the parahippocampal gyrus were excluded. The hippocampus was also divided along the long axis into an anterior and posterior portion [40]. In line with recommendations by Poppenk et al [41] we defined the anterior hippocampus as the region at or anterior to Y=-21 in MNI space. To minimise interpolation of hippocampal volumes, a mask was created in MNI space at Y=-21 that included the anterior portion of the brain, and projected to subject's native space using the inverse of the (non-linear) MNI-transformation derived from fsl_anat. The mask was thresholded at 0.5 and used to extract anterior hippocampal volumes. Hippocampal and anterior hippocampal volumes were expressed as a percentage of whole brain volume.

VBM was carried out using FSL-VBM [42], an optimised VBM protocol [43] that uses FSL tools [44]. The FSL-VBM processing pipeline was adapted for longitudinal analyses to avoid registration and interpolation biases [45]. Specifically, SIENA [46] was used to calculate the mid-space between the two (timepoint 1 and timepoint 2) T1-weighted scans for each participant. Each scan was registered to this halfway space, and then averaged. Using BET [47], a brain mask of the averaged brain was then created and realigned to each native space for both timepoints. In native space, the images were segmented with FAST, and both grey matter timepoints were then registered back to halfway space, using the halfway T1 as reference, and averaged. The averaged grey matter image for each subject was then registered to MNI space. Next, each native grey matter image was modulated using the Jacobian of the warp field produced during the non-linear transformation of the T1 image from native space to the MNI GM template. Finally, the images were smoothed with an isotropic Gaussian kernel with a sigma of 3 mm (~7 mm FWHM) and change maps (timepoint 2 – timepoint 1) created.

DTI scans were acquired with an echo planar imaging sequence (60 diffusion weighted directions, b-value 1500s/mm^2^; 5 non-diffusion weighted images, b-value 0s/mm^2^, with one b0 volume acquired in the reversed phase encoded direction) with repetition time 8900ms, echo time 91.2ms, field of view 192mm, and voxel dimensions 2.0mm isotropic. The susceptibility induced off-resonance field was estimated from a pair of b0 scans using the FSL tool topup [48]. This information was fed into the FSL tool eddy, where data was corrected for subject movement and eddy current-induced distortions [49] and for movement induced signal voids (outliers) [50]. Slices were classified as outliers and replaced if the signal was found to be more than three standard deviations from the Gaussian process predicted slice. If over 10 slices were identified as outliers within a volume, the volume was removed. If more than five volumes were removed, then the scan was excluded from analyses. DTIFit, part of FMRIB's Diffusion Toolbox, was used to fit a diffusion tensor model to the raw diffusion data, obtaining maps of FA, AD and RD. Voxel-wise analysis of DTI data was carried out using Tract Based Spatial Statistics (TBSS) [51], part of FSL [44]. TBSS projects all participants’ FA, AD and RD data onto a mean FA tract skeleton. The threshold for the mean FA skeleton was set at 0.2, resulting in a mask of 124,408 voxels, and change maps (timepoint 2 – timepoint 1) were created. Global measures of mean FA, AD and RD within the skeleton mask were calculated by averaging these values across the entire white matter skeleton.

### 2.10 Data analysis

We employed permutation-based methods for non-parametric testing for all analyses [52], using the FSL tool Permutation Analysis of Linear Models (PALM) [52] for non-voxelwise statistics and Randomise [53] for voxelwise statistics. The level of statistical significance was p < 0.05 for all analysis. For voxelwise statistics (5000 permutations), threshold-free cluster enhancement and family-wise error rate correction were used for multiple comparisons across voxels [54].

First, we examined differences in baseline demographics between included and excluded participants, and aerobic training and control groups.

Second, we examined differences between aerobic training and control groups in change scores (timepoint 2 – timepoint 1) for cardiorespiratory, PA, cognitive and MRI outcomes. When group differences were significant for any outcome, post-hoc one-sample t tests were performed for the aerobic training and control groups separately, to examine if either group displayed a significant increase or decrease in score. For analysis of cognitive outcomes, non-parametric combination (NPC) using the Fisher's combining function was used to reduce the number of comparisons by assessing the overall p-value for each cognitive domain [55]. No covariates were included in analyses of group differences. We report p-values for individual cognitive tests for descriptive purposes only.

Finally, for the aerobic training group, we examined the linear association between change in CRF over the study, and change in cognitive and MRI measures. Age, gender and education level were included as covariates in correlational analysis.

## 3 Results

### 3.1 Sample

**Fig. 1:**
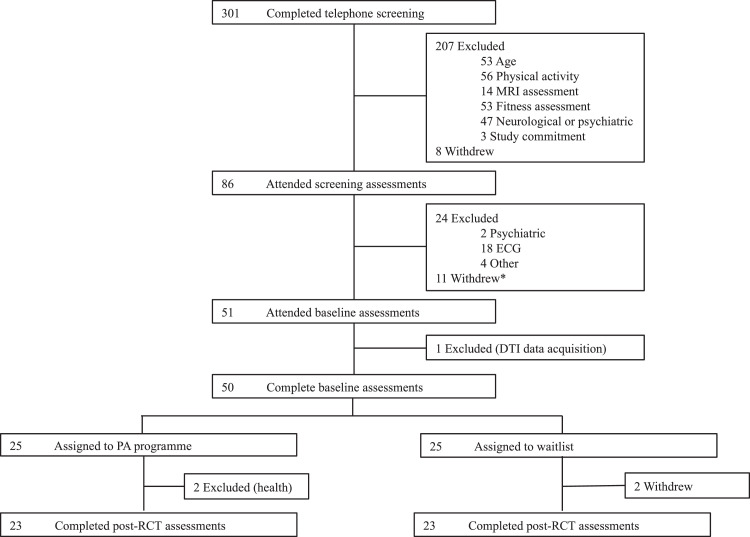
Attrition of Participants.

Three hundred and one individuals were screened over the telephone, 86 attended a screening assessment, and 51 participants completed baseline assessments and were randomized into the trial. Complete datasets for pre- and post-RCT assessments were available for 46 participants (Figure 1). Participants included in the analyses were not significantly different to those excluded analyses in terms of age, sex or education level (Supplementary Material – Table S1). Participants in the aerobic training programme attended an average of 32.1 ± 4.8 sessions (range 19 – 36), with 87% of participants completing at least 75% of sessions.

### 3.2 Group differences

Aerobic training and control groups were not significantly different in terms of age, sex or education level (Table 1).

**Table 1:** Demographics.

	Aerobic Training (N = 23)	Control (N = 23)	Cohen's d	p-value
***Demographics***				
Age (years)	65.5 ± 4.0	67.7 ± 6.0	0.42	0.436
Sex (N, % Female)	15 (65%)	14 (61%)	-0.09	0.961
Education Level	3.0 ± 1.1	3.5 ± 1.2	0.38	0.522
* corrected for multiple contrasts				

Change in VO_2_ peak was significantly greater in the aerobic training group compared with the control group (Table 2), with a significant increase in the aerobic training group (p = 0.019) and no significant change in the control group (p = 0.226). Change in moderate-to-vigorous PA was also greater in the aerobic training group compared with the control group, without significant changes in either the aerobic training group (p = 0.09) or the control group (p = 0.16). There was no significant difference between groups in change in objective PA.

**Table 2:** Group differences between aerobic training and control groups.

	Aerobic Training (n = 23)			Control (n = 23)			Group Difference in Change	
	Baseline	Follow-Up	Change	Baseline	Follow-Up	Change	Cohen's d	p
VO_2_ peak (ml.kg^−1^.min^−1^)	22.1 ± 6.4	24.5 ± 6.6	2.4 ± 4.1	21.5 ± 5.1	20.7 ± 6.1	-0.8 ± 3.2	0.88	**0.003**
MET.Minutes per week in MVPA	611.4 ± 792.9	985.4 ± 619.4	374.0 ± 685.5	1401.8 ± 1604.9	1127.3 ± 1348.4	-274.6 ± 1106.2	0.41	**0.012**
Accelerometry (average vector magnitude)	25.0 ± 6.2	24.8 ± 6.0	-0.2 ± 3.2	23.3 ± 5.7	23.2 ± 5.9	-0.1 ± 1.9	-0.03	0.457
***Executive Function***								
Digit Span: Forward	10.9 ± 2.4	11.1 ± 2.3	0.2 ± 1.7	11.1 ± 2.0	10.8 ± 1.6	-0.3 ± 1.7	0.31	0.173
Digit Span: Backward	9.2 ± 2.1	9.8 ± 2.3	0.6 ± 1.9	8.8 ± 1.7	8.8 ± 2.2	0.0 ± 2.1	0.30	0.174
Digit Span: Sequence	8.5 ± 1.8	9.2 ± 1.8	0.7 ± 1.6	8.3 ± 1.6	9.0 ± 1.6	0.8 ± 1.5	-0.08	0.424
Fluency: Category	23.5 ± 6.7	24.7 ± 6.4	1.3 ± 5.1	21.7 ± 4.1	23.6 ± 5.3	1.9 ± 4.2	-0.13	0.335
Fluency: Letter	15.2 ± 3.9	18.1 ± 4.9	2.9 ± 4.1	15.4 ± 4.1	17.9 ± 4.6	2.5 ± 4.5	0.09	0.392
TMT: B* (s)	-65.5 ± 30.7	-55.0 ± 25.6	10.5 ± 18.3	-57.9 ± 17.5	-59.4 ± 27.9	-1.5 ± 31.8	0.46	0.062
One-Back Accuracy	1.4 ± 0.1	1.4 ± 0.1	0.0 ± 0.1	1.3 ± 0.1	1.3 ± 0.1	0.1 ± 0.1	-0.30	0.154
Two-Back Accuracy	1.2 ± 0.1	1.3 ± 0.1	0.1 ± 0.1	1.2 ± 0.1	1.2 ± 0.1	0.0 ± 0.1	0.47	0.059
***Memory***								
HVLT-R: Total Recall	27.3 ± 5.1	26.6 ± 4.7	-0.7 ± 4.2	25.9 ± 5.2	25.0 ± 4.8	-1.0 ± 4.0	0.07	0.423
HVLT-R: Delayed Recall	10.0 ± 2.4	9.5 ± 2.9	-0.5 ± 2.1	8.3 ± 3.2	8.8 ± 2.5	0.4 ± 2.4	-0.41	0.104
HVLT-R: Recognition	10.6 ± 1.8	11.0 ± 1.4	0.5 ± 1.4	9.9 ± 2.0	10.5 ± 1.6	0.6 ± 2.1	-0.07	0.435
RCF: Immediate Recall	19.6 ± 5.6	23.7 ± 6.6	4.1 ± 3.9	18.2 ± 5.9	21.4 ± 5.8	3.2 ± 4.4	0.21	0.249
RCF: Delayed Recall	18.9 ± 6.1	22.6 ± 6.6	3.6 ± 4.2	16.8 ± 5.6	21.2 ± 5.6	4.4 ± 3.0	-0.22	0.238
RCF: Recognition	9.3 ± 1.7	9.7 ± 1.4	0.4 ± 1.9	9.1 ± 1.6	9.5 ± 1.6	0.4 ± 1.6	0.02	0.500
Continuous Paired Associate Errors*	-76.1 ± 44.7	-52.7 ± 45.1	23.5 ± 34.1	-74.2 ± 48.9	-77.7 ± 70.1	-3.5 ± 57.2	0.57	0.030
***Processing Speed***								
TMT: A* (s)	-24.4 ± 7.4	-25.8 ± 10.5	-1.4 ± 8.6	-25.4 ± 5.6	-28.2 ± 7.8	-2.7 ± 7.0	0.17	0.299
Digit Coding	70.0 ± 12.0	72.5 ± 13.4	2.6 ± 6.1	65.0 ± 15.6	64.7 ± 15.6	-0.3 ± 5.7	0.48	0.056
Simple: Reaction Time* (ms)	-271.9 ± 33.0	-273.8 ± 26.0	-1.9 ± 25.0	-276.9 ± 52.3	-274.8 ± 33.2	2.1 ± 39.6	-0.12	0.341
Choice: Reaction Time* (ms)	-300.8 ± 29.7	-300.6 ± 28.6	0.3 ± 24.5	-308.3 ± 60.2	-312.5 ± 46.3	-4.2 ± 37.4	0.14	0.326
Simple: Movement Time* (ms)	-232.1 ± 54.0	-239.3 ± 63.8	-7.2 ± 52.8	-250.4 ± 65.8	-246.0 ± 45.9	4.4 ± 41.0	-0.24	0.209
Choice: Reaction Time* (ms)	-249.9 ± 52.5	-264.9 ± 66.3	-15.0 ± 61.7	-256.5 ± 72.2	-274.5 ± 79.1	-18.0 ± 69.8	0.04	0.442
Detection Speed* (log_10_ ms)	-2.5 ± 0.1	-2.6 ± 0.1	0.0 ± 0.1	-2.6 ± 0.1	-2.5 ± 0.1	0.0 ± 0.0	-0.46	0.068
Identification Speed* (log_10_ ms)	-2.7 ± 0.1	-2.7 ± 0.	0.0 ± 0.0	-2.7 ± 0.1	-2.7 ± 0.05	0.0 ± 0.0	-0.15	0.309
***MRI Measures***								
Left Hippocampus (%)	0.245 ± 0.023	0.244 ± 0.026	-0.001 ± 0.005	0.236 ± 0.034	0.236 ± 0.032	0.000 ± 0.009	-0.10	0.367
Right Hippocampus (%)	0.253 ± 0.026	0.254 ± 0.031	0.001 ± 0.008	0.242 ± 0.034	0.242 ± 0.035	-0.001 ± 0.008	0.25	0.197
Left Anterior Hippocampus (%)	0.127 ± 0.015	0.127 ± 0.015	0.000 ± 0.003	0.125 ± 0.019	0.126 ± 0.018	0.001 ± 0.005	-0.13	0.326
Right Anterior Hippocampus (%)	0.141 ± 0.018	0.142 ± 0.021	0.001 ± 0.006	0.137 ± 0.021	0.137 ± 0.021	0.000 ± 0.006	0.13	0.183
Global FA	0.478 ± 0.013	0.477 ± 0.013	-0.001 ± 0.003	0.475 ± 0.015	0.474 ± 0.015	-0.001 ± 0.003	-0.07	0.403
Global AD (x10^3^)	1.071 ± 0.020	1.071 ± 0.024	0.000 ± 0.007	1.075 ± 0.025	1.075 ± 0.022	0.000 ± 0.007	-0.02	0.470
Global RD (x10^3^)	0.482 ± 0.019	0.483 ± 0.020	0.001 ± 0.003	0.487 ± 0.024	0.488 ± 0.022	0.001 ± 0.004	0.06	0.423

Values are mean ± standard deviation.

⁎ reverse scored so that higher scores indicate better performance. N=24 for control group for accelerometry analysis.

There were no significant differences between groups for change in executive function (Fisher NPC p = 0.113), memory (p = 0.386) or processing speed (p = 0.442). No significant differences between groups were observed for left or right hippocampal or anterior hippocampal volume, or detected with VBM. Change in FA, AD and RD was not significantly different between groups in either global or voxel-wise analysis.

### 3.3 Correlations

Change in fitness was not associated with change in executive function (p = 0.094, positive), memory (p = 0.193, positive) or processing speed (p = 0.151, negative). Change in fitness was not associated with hippocampal volume (left r = 0.24, p = 0.127; right r = 0.14, p = 0.227), anterior hippocampal volume (left r = -0.16, p = 0.470; right r = 0.244, p = 0.101), global FA (r = -0.23., p = 0.129), AD (r = -0.24, p = 0.133) or RD (r = 0.02, p = 0.464). VBM and TBSS analyses were also not significant.

## 4 Discussion

We examined the effects of a 12-week aerobic training intervention on CRF, white matter microstructure and cognition in healthy older adults. Although the intervention resulted in improvements in CRF and self-reported moderate-intensity PA, there were no significant differences between the aerobic training and control groups in cognitive and white matter measures. Furthermore, change in CRF over the intervention was not found to be significantly associated with change in cognitive or DTI measures within the aerobic training group.

Our null findings reflect a mixed literature concerning whether aerobic training interventions can improve cognition and measures of brain structure in healthy older adults. With regard to cognition, there are conflicting findings not only between individual studies, but also between meta-analyses. For example, early meta-analyses of interventional studies reported improvements across executive, controlled processing, spatial and speed tasks [6], and attention and processing speed, executive function and memory [7]. However, a subsequent systematic review and meta-analysis found *no* effect of aerobic PA interventions across multiple cognitive domains including recognition, immediate recall, delayed recall, verbal fluency, reasoning, working memory, attention, processing speed [10]. Similarly, a 2015 Cochrane review concluded that there is *no* evidence that aerobic PA interventions have *any* benefit in cognitively healthy older adults across eleven cognitive domains spanning attention, memory, perception, executive functions, cognitive inhibition, cognitive speed and motor function [11]. More recently, though, Barha et al reported that aerobic and multi-component training interventions did lead to improvements in executive function, word fluency, visuospatial function, processing speed and global cognition; with multi-component training, but not aerobic training, also enhancing episodic memory [9]. Finally, Northey et al reported that interventions that spanned aerobic, resistance and multi-component approaches resulted in improvements to attention, executive function, memory and working memory [8].

MRI studies examining grey matter volumes have been similarly variable. For example, individual studies examining change in hippocampal volume between aerobic training and control groups have reported both positive [17,56] and null [18], [19], [20] findings. Although a meta-analysis of five studies showed that aerobic training was associated with a significant increase in both left and right hippocampal volumes compared with control conditions, a meta-analysis of six studies found no effect on total hippocampal volume [57]. The picture is no more clear for other brain regions [18,21,22]. While fewer studies have examined white matter integrity, to the best of our knowledge. ours is now the fourth to report no significant effect of aerobic training compared with control conditions on DTI metrics, with no study reporting significant group effects [23,25,58].

There are many factors that may have contributed to our, and others’, null findings. First, our sample size was limited. We hope, though, that our study can contribute to future meta-analyses including larger numbers of participants.

Second, our participants comprised cognitively *healthy* older adults, so it is possible a ceiling effect may have been present. Interestingly, there is some evidence to support beneficial effects of PA interventions for Mild Cognitive Impairment and dementia [59,60], although other trials have reported null effects [61]. In addition, participants with uncontrolled hypertension or a history of major vasculature problems were excluded from the study, limiting vascular risk factors in the sample. Vascular risk factors are associated with MRI indices throughout the lifespan [62,63], and targeting individuals with risk factors may be an effective strategy for interventional studies [64]. In addition, average baseline MET.Minutes per week exceeded the threshold of 500 MET.Minutes per group that is the basis of PA guidelines. This is despite all participants self-reporting fewer than 60 minutes per week of PA that raises their heart rate in the screening assessment. There are many possible reasons for this discrepancy – including differences in assessment methods and delay between the initial screening assessment and baseline assessments. Interestingly, high MET.Minutes per week values at baseline, particularly within the waitlist control group, often arose from participants reporting high levels of “heavy work around the house (such as washing windows, cleaning gutters)” and “heavy gardening (such as digging, raking)” (Supplementary Material Table S2). These activities are classified as MVPA in the CHAMPS questionnaire, but may have been performed at a level that was not sufficient to raise participants’ heart rates for prolonged periods of time.

Third, the type of PA may have been insufficient to result in cognitive gains. To date, there has been much focus on the role improved CRF plays in mediating the link between PA and cognition [65], and our PA intervention was designed with the aim of improving CRF. Notably, though, our intervention did not lead to improvements in cognition despite resulting in improvements in CRF. Similarly, 9 of the 12 trials included in the Cochrane review of cognitive studies reported that the PA intervention resulted in increased CRF, and analysis of this subgroup of studies showed that this improvement in fitness did not coincide with improvements in any cognitive domains assessed [11]. Furthermore, change in CRF over the intervention was not found to be significantly associated with cognitive performance post-intervention, in agreement with a meta-regression that failed to find a significant relationship between fitness and cognitive effect sizes [66]. Taken together with the wider literature, our results may indicate that the association between PA levels and cognition demonstrated by epidemiological studies may be driven by factors often associated with PA other than solely CRF, such as improvement in strength, social engagement, cognitive stimulation, and a range of other health benefits (e.g. BMI, blood pressure, depression and sleep). Interestingly, both pedal and e-bike outdoor cycling were recently shown to improve executive function over 8-weeks, which too suggests beneficial effects of PA are not solely exerted through CRF [67]. Furthermore, a dance intervention that combines physical, cognitive and social engagement was found to result in increases in FA within the fornix [24]. Finally, the results of the Finnish Geriatric Intervention Study to Prevent Cognitive Impairment and Disability (FINGER) study indicated that a 2-year multi-domain intervention (diet, exercise, cognitive training, vascular risk monitoring) could improve or maintain cognitive functioning in at-risk older adults [64].

Fourth, although cycling has been shown to enhance cognitive performance during and after acute bouts of exercise [68], the duration of the intervention may not have been a factor. Rather than relatively short-term interventions examining if PA can *improve* cognition and MRI measures in healthy older adults, studies aimed at *preventing* or *slowing* cognitive decline may have more success.

In summary, we found no evidence that a 12-week PA intervention led to improvements in cognitive functioning or markers of white matter microstructure in older adults. Multi-factorial longer-term intervention studies, enrolling participants at risk for developing or displaying cognitive impairment, may yield more encouraging results.

## Appendix Supplementary materials

Image, application 1
