# Supplementary material for: The effects of an aerobic training intervention on cognition, grey matter volumes and white matter microstructure
Source: Physiol Behav. 2020 Sep 1;223:112923. doi: 10.1016/j.physbeh.2020.112923 (PMC7378567; doi:10.1016/j.physbeh.2020.112923)
Supplement: Supplementary file 1 [file mmc1.docx]

**Text S1. Accelerometry Analysis Protocol**

Accelerometry data was processed following the methods developed by the UK Biobank Expert Working Group. To ensure different devices provided a similar output under similar conditions, the acceleration signals were calibrated to local gravity using the procedure described by van Hees and colleagues (van Hees et al., 2014). For calibration, we identified stationary periods in ten-second windows where all three axes had a standard deviation of less than 13.0 m*g.* These stationary periods were then used to optimise the gain and offset for each axis (6 parameters) to fit a unit gravity sphere using ordinary least squares linear regression. After calibration, the sample level Euclidean norm of the acceleration was calculated in x/y/z axes, and machine noise removed using a fourth order Butterworth low pass filter with a cutoff frequency of 20Hz. In order to separate out the activity-related component of the acceleration signal, one gravitational unit was removed from the vector magnitude, with remaining negative values truncated to zero (da Silva et al., 2014; Sabia et al., 2014).

To describe the overall level and distribution of physical activity intensity, the sample level data was combined into five-second epochs for summary data analysis, maintaining the average vector magnitude value over the epoch. Non-wear time, defined as consecutive stationary episodes lasting for at least 60 minutes, was removed (Sabia et al., 2014; van Hees et al., 2011). Non-wear data segments were imputed using the average of similar time-of-day vector magnitude data points with one minute granularity on different days of the measurement, as in previous studies (van Hees et al., 2011). A physical activity outcome variable, average vector magnitude, was constructed by averaging all worn and imputed values. No participants were excluded due to faulty device data, defined as >1% of values exceeding the sensors ± 8g range before/after calibration. We excluded data from participants who had less than three days (72 hours) of data or who did not have data in each one-hour period of the 24-hour cycle (N=1).

**Table S1. Missing Data**

|  | Included (N = 46) | Missing (N = 5) | Cohen’s d | p-value* |
| --- | --- | --- | --- | --- |
| ***Demographics*** |  |  |  |  |
| Age (years) | 66.6 ± 5.2 | 68.6 ± 8.5 | 0.37 | 0.751 |
| Sex (N, % Female) | 29 (63%) | 4 (80%) | 0.35 | 0.828 |
| Education Level | 3.3 ± 1.2 | 4.2 ± 0.8 | 0.82 | 0.270 |

* corrected for multiple contrasts

**Table S2. Physical Activity at Baseline By CHAMPS Question**

|  | Aerobic Training  (n = 23) | Control  (n = 25) |
| --- | --- | --- |
| 7. Dance (such as square, folk, line, ballroom) (do not count aerobic dance here)? | 5.87 ± 27.53 | 41.09 ± 133.14 |
| 9. Play golf, carrying or pulling your equipment (count walking time only)? | 0.00 ± 0.00 | 29.35 ± 137.65 |
| 14. Play singles tennis (do not count doubles)? | 0.00 ± 0.00 | 0.00 ± 0.00 |
| 15. Play doubles tennis (do not count singles)? | 0.00 ± 0.00 | 0.00 ± 0.00 |
| 16. Skate (ice, roller, in-line)? | 0.00 ± 0.00 | 0.00 ± 0.00 |
| 19. Do heavy work around the house (such as washing windows, cleaning gutters)? | 168.26 ± 231.83 | 315.00 ± 499.87 |
| 21. Do heavy gardening (such as spading, raking)? | 120.00 ± 303.37 | 331.30 ± 466.86 |
| 23. Work on your car, truck, lawn mower, or other machinery? | 33.26 ± 138.04 | 125.22 ± 274.83 |
| 24. Jog or run? | 9.13 ± 42.83 | 9.13 ± 42.83 |
| 25. Walk uphill or hike uphill (count only uphill part)? | 97.83 ± 142.71 | 156.52 ± 330.65 |
| 26. Walk fast or briskly for exercise (do not count walking leisurely or uphill)? | 52.50 ± 123.85 | 148.37 ± 295.63 |
| 29. Ride a bicycle or stationary cycle? | 52.17 ± 120.40 | 117.39 ± 258.51 |
| 30. Do other aerobic machines such as rowing, or step machines (do not count treadmill or stationary cycle)? | 6.52 ± 30.59 | 6.52 ± 30.59 |
| 31. Do water exercises (do not count other swimming)? | 3.91 ± 18.35 | 0.00 ± 0.00 |
| 32. Swim moderately or fast? | 35.87 ± 112.49 | 0.00 ± 0.00 |
| 33. Swim gently? | 7.83 ± 25.36 | 21.52 ± 67.49 |
| 36. Do aerobics or aerobic dancing? | 4.57 ± 21.41 | 25.11 ± 78.74 |
| 37. Do moderate to heavy strength training (such as hand-held weights of more than 5 lbs., weight machines, or push-ups)? | 5.87 ± 27.53 | 26.41 ± 99.00 |
| 38. Do light strength training (such as hand-held weights of 5 lbs. or less or elastic bands)? | 7.83 ± 25.36 | 48.91 ± 211.03 |
| 40. Play basketball, soccer, or racquetball (do not count time on sidelines)? | 0.00 ± 0.00 | 0.00 ± 0.00 |

**References**

da Silva, I.C., van Hees, V.T., Ramires, V. V, Knuth, A.G., Bielemann, R.M., Ekelund, U., Brage, S., Hallal, P.C., 2014. Physical activity levels in three Brazilian birth cohorts as assessed with raw triaxial wrist accelerometry. Int. J. Epidemiol. 43, 1959–68.

Sabia, S., van Hees, V.T., Shipley, M.J., Trenell, M.I., Hagger-Johnson, G., Elbaz, A., Kivimaki, M., Singh-Manoux, A., 2014. Association between questionnaire- and accelerometer-assessed physical activity: the role of sociodemographic factors. Am. J. Epidemiol. 179, 781–90.

van Hees, V.T., Fang, Z., Langford, J., Assah, F., Mohammad, A., da Silva, I.C.M., Trenell, M.I., White, T., Wareham, N.J., Brage, S., 2014. Autocalibration of accelerometer data for free-living physical activity assessment using local gravity and temperature: an evaluation on four continents. J. Appl. Physiol. 117, 738–44.

van Hees, V.T., Renström, F., Wright, A., Gradmark, A., Catt, M., Chen, K.Y., Löf, M., Bluck, L., Pomeroy, J., Wareham, N.J., Ekelund, U., Brage, S., Franks, P.W., 2011. Estimation of daily energy expenditure in pregnant and non-pregnant women using a wrist-worn tri-axial accelerometer. PLoS One 6, e22922.
